# Cloud-based large-scale curation of medical imaging data using AI segmentation

**DOI:** 10.21203/rs.3.rs-4351526/v1

**Published:** 2024-05-03

**Authors:** Vamsi Krishna Thiriveedhi, Deepa Krishnaswamy, David Clunie, Steve Pieper, Ron Kikinis, Andrey Fedorov

**Affiliations:** 1 Brigham and Women’s Hospital, Boston, MA; 2 PixelMed Publishing, Bangor, PA; 3 Isomics Inc., Cambridge, MA

**Keywords:** cloud computing, AI, image segmentation, computed tomography, radiomics

## Abstract

Rapid advances in medical imaging Artificial Intelligence (AI) offer unprecedented opportunities for automatic analysis and extraction of data from large imaging collections. Computational demands of such modern AI tools may be difficult to satisfy with the capabilities available on premises. Cloud computing offers the promise of economical access and extreme scalability. Few studies examine the price/performance tradeoffs of using the cloud, in particular for medical image analysis tasks. We investigate the use of cloud-provisioned compute resources for AI-based curation of the National Lung Screening Trial (NLST) Computed Tomography (CT) images available from the National Cancer Institute (NCI) Imaging Data Commons (IDC). We evaluated NCI Cancer Research Data Commons (CRDC) Cloud Resources - Terra (FireCloud) and Seven Bridges-Cancer Genomics Cloud (SB-CGC) platforms - to perform automatic image segmentation with *TotalSegmentator* and *pyradiomics* feature extraction for a large cohort containing >126,000 CT volumes from >26,000 patients. Utilizing >21,000 Virtual Machines (VMs) over the course of the computation we completed analysis in under 9 hours, as compared to the estimated 522 days that would be needed on a single workstation. The total cost of utilizing the cloud for this analysis was $1,011.05. Our contributions include: 1) an evaluation of the numerous tradeoffs towards optimizing the use of cloud resources for large-scale image analysis; 2) *CloudSegmentator,* an open source reproducible implementation of the developed workflows, which can be reused and extended; 3) practical recommendations for utilizing the cloud for large-scale medical image computing tasks. We also share the results of the analysis: the total of 9,565,554 segmentations of the anatomic structures and the accompanying radiomics features in IDC as of release v18.

## Introduction

Medical imaging researchers are confronted with the ever-increasing sizes of datasets. Such datasets may be produced in the course of clinical trials or as part of other dedicated data collection and sharing activities. Gaining insights from the images often requires annotation of the regions of interest and post-processing to extract quantitative information^1^. These quantitative measurements can then be used to evaluate putative imaging biomarkers, conduct multi-omics analyses by fusing imaging with other sources of data characterizing the disease, and conduct population studies^2^, among other applications. Manual annotation of the regions of interest in the images is often prohibitive due to the sheer size of the datasets. Even expert annotations may have deficiencies due to inter-reader variability, differences in the reader training and conventions defining the segmented region. With the recent advances in automated annotation tools, there is emerging evidence that Artificial Intelligence (AI)-based curation can be an effective tool for large-scale image annotation. However, applying these automated methods efficiently to massive datasets remains a key challenge. This study investigates the use of cloud-based computational resources and their potential to democratize access to AI-based large-scale annotation.

National Lung Screening Trial (NLST)^3,4^ is one of the largest publicly available cancer screening imaging datasets. It contains over 10 terabytes of Computed Tomography (CT) images for over 26 thousand patients. The NLST collection is rather heterogeneous, containing screening CT images collected at over 30 cancer centers using scanners from 4 manufacturers. NLST images are accompanied by rich clinical metadata, including some of the attributes describing imaging findings (e.g., CT slice containing abnormality’s greatest diameter), allowing investigation of various secondary hypotheses, as demonstrated by Zeleznik et al.^5^ among others.

The original NLST collection does not contain any volumetric segmentations of either abnormalities or anatomic structures, limiting its secondary analysis. As discussed elsewhere^6^, volumetric segmentations of the regions of interest - both anatomic organs and malignancies - significantly improve the utility of the dataset, since it is a common preprocessing step in the more complex analysis pipelines that investigate the utility of imaging biomarkers in various applications, and can be used for extracting various image-based features. The availability of segmentations enables investigation of secondary hypotheses associating clinical findings with the image content, and improves the searchability of the data, allowing the building of cohorts based on the presence of certain anatomy in the image. Furthermore, the application of a segmentation model to a large dataset can be used to evaluate its generalizability and robustness. Recently, NLST became available within the National Cancer Institute (NCI) Imaging Data Commons (IDC)^7^. By hosting all of its data within the cloud IDC aims to make it easier to analyze its content using scalable cloud resources.

*TotalSegmentator*^8^ is a recently introduced Deep Learning (DL) model designed to segment up to 104 anatomic structures (but not the tumors) from CT scans. In this study, we demonstrate how cloud computing resources can be used to apply *TotalSegmentator* to enrich the NLST collection. With the NLST collection containing over 200,000 CT scans, sequential application of the developed workflow to NLST on a conventional workstation would take well over a year. Parallel computing solutions such as GPU clusters are available at many labs, and may be available for free or at a low cost to affiliated researchers. But institutional compute resources vary widely in terms of access, performance, and system configurations. They also require sustained investment for maintenance and the hardware must be updated periodically to remain current and there may be contention between groups within a lab for access to compute resources. Many institutions that currently or historically have operated their own high performance computing installations are reconsidering their investments in data centers and support personnel. These considerations create a strong motivation for better understanding the options for scalable and predictably available cloud resources to expedite the analysis. In the cloud scenario, anyone with financial resources can perform this analysis, without gaining permission from the owners of any institutional resources or ensuring that appropriate hardware, drivers, and other installation configurations are compatible with the *TotalSegmentator* requirements. *TotalSegmentator* is a sophisticated AI application that not only adds value to cancer imaging data by enriching it with the annotations of anatomic structures, but also can serve as a proxy to assess the performance and economics of cloud computing. We fully expect that the per-segment radiomic feature quantitative data that resulted from our analysis could be used together with NLST clinical data to explore cancer research hypotheses, but this paper does not explore that type of analysis.

As we describe more fully below, cloud computing refers generally to large collections of computer resources housed in warehouse-sized data centers and made available for short or long-term rental as an alternative to the more traditional so-called on-premises model of purchasing and operating computers. There are many dimensions to compare these approaches, such as ease of use, return on investment, operational control, data security, and scalability. In this study, we focus on the practicality and cost of the cloud computing option because to our knowledge, these factors have not been previously reported for large scale AI analysis of publicly available cancer imaging data and our experience may help guide future investigation in this area.

We present a detailed investigation of the development and optimization of a computational workflow to perform NLST segmentation efficiently - both in terms of processing time and costs - using the Google Cloud Platform and the components of the NCI Cancer Research Data Commons (CRDC)^9^ - a cloud-based data science infrastructure that provides secure access to a large, comprehensive, and expanding collection of cancer research data.

We describe a series of experiments to refine the workflow and guide the optimization choices, ultimately leading to the successful application of the workflow to the entire cohort of images suitable for the analysis. This study demonstrates the potential of the cloud to improve our ability to scale computationally demanding image analysis tasks and apply the latest advances in DL to large datasets at acceptable costs. As we demonstrate in this study, cloud-based computing resources make it possible to reduce processing time by many orders of magnitude as compared to sequential analysis at a rather affordable cost. We evaluated various tradeoffs affecting the processing time and the monetary cost of the computation, culminating in practical recommendations for conducting large image analysis experiments using the cloud.

## Materials and Methods

### TotalSegmentator processing pipeline

*TotalSegmentator*^8^ version 1 is a recently introduced model based on the nnU-Net framework^10^ that can be used to segment 104 anatomic structures (27 organs, 59 bones, 10 muscles, and 8 vessels) from CT examinations. The model was trained and evaluated on datasets collected at the University Hospital Basel (1024 and 4004 CT volumes, respectively, for the training and evaluation dataset), containing scans for patients with a range of pathologies, with most images acquired using equipment from the same manufacturer. The original evaluation of the *TotalSegmentator* model established high segmentation accuracy on the dataset used for model development. It examined correlations of CT attenuation and segmented structure volume with subject age in the test cohort. The model is shared via a publicly available GitHub repository, accompanied by a command-line tool that can be used to apply the model to an input image stored in DICOM or NIfTI formats.

**Figure 1:**
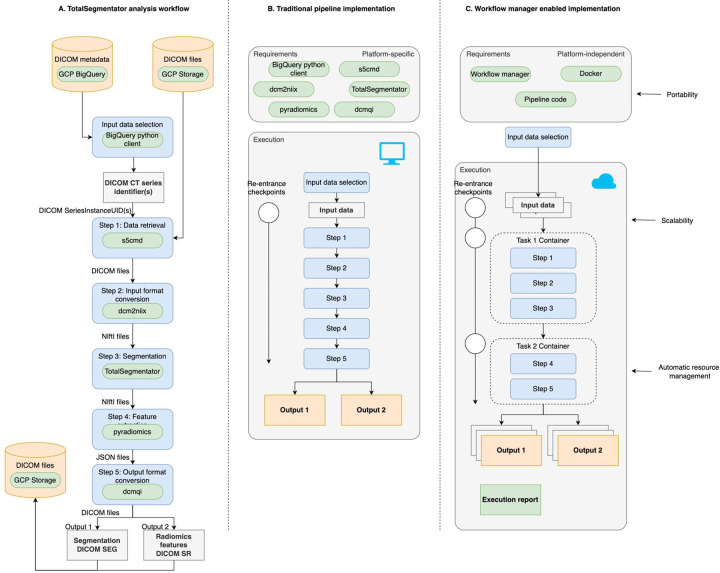
Summary of the TotalSegmentator analysis workflow implementation (based on the concept in Figure 1 of Wratten et al.^11^). A: conceptual outline of the analysis workflow indicating the flow of data and processing steps. B: traditional pipeline implementation suitable for execution in a pre-configured computational environment. C: workflow manager-enabled implementation simplifies the execution of the pipeline on cloud resources (“twoVM” configuration of the workflow shown).

Application of the TotalSegmentator to the IDC NLST collection involves the following steps, also shown in Figure 1A:

1. **Input data selection** CT studies often contain multiple acquisitions, which may include projection or localizer images not suitable for volumetric analysis. Furthermore, some CT acquisitions may not be possible to reconstruct into coherent 3D volumes due to, for example, incomplete or corrupted data. In general, only a subset of the CT series would be compatible with *TotalSegmentator* analysis.
2. **Step 1: Data retrieval** DICOM files corresponding to the CT series being analyzed need to be efficiently retrieved from IDC cloud buckets.
3. **Step 2: Input format conversion** The NLST collection was shared in its native DICOM format. Although TotalSegmentator can read well-formatted CT volumes directly from DICOM, to account for variability in the NLST dataset we chose to use *dcm2niix*^12^, a more robust and fault-tolerant conversion tool and provide NIfTI files in a consistent format to TotalSegmentator.
4. **Step 3: Segmentation** The application of TotalSegmentator to a suitable volumetric CT series requires appropriate hardware configuration with a sufficiently powered GPU or CPU and enough memory.
5. **Step 4: Radiomics feature extraction** Basic radiomics features (e.g., volume, mean attenuation, or sphericity) can be used to summarize the basic characteristics of the segmentations, which in turn can be useful in identifying failure cases and outliers. Radiomics features also provide value in enabling secondary analysis of the NLST collection, for example, to investigate acquisition-, age- or sex-related patterns, as was done by Wasserthal et al.^13^.
6. **Step 5: Output format conversion** For consistency with IDC practices, we convert the results into DICOM Segmentation format in order to achieve interoperability with IDC tools to archive and visualize the results of the analysis, extract metadata as necessary for the exploration and searching of the analysis results, and achieve FAIR representation of such data for archival purposes^7^.

Given the processing steps above, and the size of the NLST collection, there is strong motivation to parallelize analysis and reduce processing time. In this study, we address this challenge by using cloud computing resources.

### Ethics declarations

This study analyzed Computed Tomography images of human subjects contained within the publicly available National Lung Screening Trial collection (NLST)^3,4,14^. Given that the study involves de-identified publicly available data, it qualifies as NIH Exempt Human Subject Research under Exemption 4.

### Cloud computing for large-scale analysis

Following Weinman^15^, cloud is defined by the five salient characteristics: common infrastructure, location-independence, online accessibility, utility pricing, and on-demand resources. In practice, there is a steep learning curve associated with the common infrastructure provided by cloud providers. There are also practical concerns related to achieving utility pricing along with on-demand scalability.

First, at the time of this writing, commercial cloud providers do not have mechanisms to enable users to stop usage after a user-defined budget is reached. Instead, there are complex quotas for individual resources and alert mechanisms to provide automatic notifications. Those alerts, however, may arrive with significant delays and do not allow bound spending.

Second, estimation of costs, in the general case, can be very difficult. The use of the cloud typically involves a broad range of services with a complex cost structure. Worse, hitting a quota such as a limit on API requests over a given time period on a specific resource may artificially slow down certain computations, even while other parts of the workflow that depend on these results continue to operate and incur costs (e.g. a VM may be idle, but incurring costs, while waiting for an API to become available again). Such interdependencies between quotas and overall costs can make it difficult or impossible to use data from limited test cases that do not reach the quota limit to estimate the costs of running larger jobs where such quota limit is reached.

Instead of using the public cloud services directly, our approach used the infrastructure built on top of the generic capabilities provided by the cloud providers to support scientific analysis workflows. We implemented the processing pipeline described earlier as a portable workflow compatible with the CRDC Terra (available under FireCloud within CRDC; further referred to as Terra for the sake of brevity) and Seven Bridges Cancer Genomics Cloud (SB-CGC) cloud resources (CRDC CRs)^9^. CRDC CRs are analytical components of CRDC that aim to simplify access to cloud-ready tools and enable cloud-based analysis of the data available in CRDC. Among the three CRDC CRs available, we selected Terra and SB-CGC due to their support of scalable workflow execution. These platforms were developed to run bioinformatics pipelines in an interoperable and reproducible way and to simplify use of the storage, access control, and logging functionality of the underlying cloud resources available from the providers. Both Terra and SB-CGC simplify the use of the cloud by allocating compute resources on behalf of the user, running the code, providing a place to organize the data, and releasing the resources immediately after a job is finished.

#### Terra

Terra^16^ is a managed service developed by the Broad Institute in collaboration with Microsoft and Verily for executing bioinformatics pipelines. Terra is deployed under many rebranded versions resulting from different partnerships with various organizations under the National Institute of Health (NIH) demonstrating the popularity of the platform: FireCloud^17^ with the National Cancer Institute (NCI), AnVIL^18^ with the National Human Genome Research Institute (NHGRI), BioData Catalyst^19^ with National Heart, Lung, and Blood Institute (NHLBI), eLwazi^20^ Open Data Science Platform for health applications in Africa with National Institution of Biomedical Imaging and Bioengineering (NIBIB).

Terra workflows are defined using Workflow Definition Language (WDL). WDL workflows include a declaration of the input files (“input”, desired virtual machine specifications, Docker image(s) encapsulating individual processing steps (“Docker” parameter of the “runtime” configuration), the commands to run inside the Docker container(s), and the expected output files. Moreover, a workflow may contain more than one task, each of which can run in its own Docker container on its own virtual machine, enabling efficient use of compute resources. Workflow execution is parameterized via a data table containing references to inputs. The data table is populated with the URLs referencing the outputs when workflow execution is completed. WDL^21^ is maintained by the OpenWDL open source community, and is “programming language lite”, meaning it offers a subset of traditional programming language features, and it is not tied to a specific programming environment and is independent of the underlying execution platform.

While the workflows can be executed on any high-performance computing cluster or a cloud provider, Terra is currently deployed with the Google Cloud Platform (GCP) as the only cloud provider for allocating compute resources. Microsoft Azure as an option is currently in public preview at the time of writing this manuscript. Terra parses the instructions in WDL using the Cromwell workflow engine^21^, which assigns the jobs using Google Cloud Lifesciences API^22^ and monitors their status. Terra provides a high level of flexibility in configuring the virtual machines for executing the individual tasks of the workflow, allowing the user to customize the number of virtual CPUs, RAM, and GPUs.

#### Seven Bridges Cancer Genomics Cloud

SB-CGC^23^ is a cloud-based research platform developed by Velsera under a contract from NCI. SB-CGC relies on Common Workflow Language (CWL) for supporting workflows. CWL workflows consist of *tools* - scripts dedicated to run a specific task in a Docker container. Workflows consist of tools composed in a linear or scattered manner. CWL workflows enable reproducibility by declaring the inputs and expected outputs, and the characteristics of the runtime environment. Similar to WDL, CWL is an open source initiative^24^, is vendor-neutral, not tied to a specific programming environment, and is independent of the underlying execution platform. CWL tools or workflows can run on a laptop, on a high-performance computing cluster, or any cloud provider^25^.

SB-CGC, at the time of writing this manuscript, offers integration with Amazon Web Services (AWS) as the primary cloud provider for allocating compute resources, with many pre-configured AWS VM instance types (customization of the VM by the user is not possible). While GCP is also supported, the VM instances are limited to the N1 family and do not include any GPU-enabled configurations. SB-CGC implementation of CWL can also be deployed locally or in a hybrid mode^26^.

Seven Bridges Genomics (SB) established partnerships with two entities besides CRDC: CAVATICA^27^ with the Center for Data Driven Discovery in Biomedicine at Children’s Hospital of Philadelphia, and BioData Catalyst^19,28^ with National Heart, Lung, and Blood Institute (NHLBI).

#### NCI Imaging Data Commons

IDC (https://imaging.datacommons.cancer.gov/) is a cloud-based environment providing public access to a broad range of cancer imaging data along with the tools to simplify the use of this data^7^. Along with Genomics, Proteomics and Canine Data Commons, IDC is a data node component within CRDC. As of version v18 released April 2024, IDC hosts over 66TB of publicly available cancer imaging and image-derived data. IDC hosts the entire NLST collection as DICOM files available for download using standard S3 API from cloud storage buckets, with the content mirrored between Google Cloud Platform (GCP) and Amazon Web Services (AWS) buckets. To support search of the data, IDC hosts DICOM metadata extracted from the NLST DICOM images in Google BigQuery - a scalable query and analysis service based on Google Dremel technology^29^. BigQuery organizes data in tables that can be queried via a dialect of the Standard Query Language (SQL).

### Analysis workflow development

In the following, we first discuss the approach to implementation of the individual steps of the workflow, which correspond to the steps of the processing pipeline discussed earlier. Next, we detail the process for developing and refining the cloud-based implementation of the workflow. WDL and CWL workflow systems utilized by the Terra and SB-CGC platforms, respectively, aim to simplify the scaling of the analysis to a large number of samples. The development of WDL/CWL workflows does require upfront investment to containerize individual processing steps and formalize the pipeline according to the specifications. Once the workflow is developed, however, the user is shielded from its complexities and can enjoy portability, scalability, and improved robustness of the analysis^11,30^, as illustrated in Figure 1C. In the following we discuss the details of the implementation for the individual steps of the workflow. Note that versions of all of the packages and tools used by the workflow are provided for completeness, but are also fixed in the Dockerfiles. Dockerfiles are available in *CloudSegmentator* workflows/TotalSegmentator/Dockerfiles, which contains all of the source code artifacts discussed in this report (here and in the following references to the source code components, we utilize paths relative to the root of the repository of CloudSegmentator release 1.2.0^31^).

#### Data selection

In this step we select the images (DICOM series) suitable for the analysis by *TotalSegmentator*. Since NLST contains not only CT data, but also other modalities such as Slide Microscopy and annotations for 571 of studies introduced by^32^, we first filtered for CT modality only and ignored any series that was either a localizer or containing less than 50 slices (this threshold was selected somewhat arbitrarily to eliminate potentially problematic series). To select series that could be reconstructed into volumes, we excluded those that had inconsistent values for ImageOrientationPatient, PixelSpacing, SliceThickness, spatially overlapping slices, or inconsistent distance between slices. The latter utilized the threshold of 0.01 mm for the maximum allowed inter-slice distance difference. The query was applied to the BigQuery dicom_all table of the IDC v17 release, producing the list of SeriesInstanceUIDs corresponding to the DICOM series deemed suitable for the analysis. The complete query is available in *CloudSegmentator* workflows/TotalSegmentator/sqlQueries/nlstCohort.sql.

#### Step 1: Data retrieval

NLST CT images are stored in the GCP/AWS storage buckets and must be downloaded for conversion. While downloading is a trivial step, it must be done efficiently due to the very large overall size of data that must be analyzed. For transferring DICOM files from the cloud bucket we used the *idc-index* python package (https://pypi.org/project/idc-index) to download DICOM data from IDC buckets given the list of SeriesInstanceUIDs. *idc-index* wraps the *s5cmd* command line tool (https://github.com/peak/s5cmd) for efficiently downloading data via S3 API.

#### Step 2: Input format conversion

DICOM CT files corresponding to the individual series were converted into NIfTI format using the open-source *dcm2niix* converter^12^ v1.0.20230411. Conversion was configured to compress the output NIfTI file (“-z y” option) and merge 2D slicers from the same series regardless of echo, exposure, etc (quoted from the documentation) (“-m y”). We selected *dcm2niix* due to its demonstrated robustness, long history of development and user support.

#### Step 3: Segmentation

*TotalSegmentator* v1.5.6 was applied to the output of the previous step. We chose to use v1 as opposed to the more recent v2 of the model for two reasons. First, v2 was not yet released at the time we started our initial experiments. Second, and more importantly, v2 introduced restrictions on the use of the model for commercial applications. Our goal was to make the results of our study available with minimal restrictions. *TotalSegmentator* can be used on both CPU and GPU platforms, allowing us to compare the cost-performance implications for VMs with or without GPUs. Inference results in NIfTI format were compressed using *lz4* (https://github.com/lz4/lz4).

#### Step 4: Radiomics feature extraction

Image feature extraction was included as a workflow step to support evaluation and checks of the segmentation results, and to support and simplify other uses that only require segmentation-extracted features. For this purpose, we utilized *pyradiomics*^33^ v3.0.1 configured to extract 28 first-order and shape features listed in *CloudSegmentator* workflows/TotalSegmentator/resources/radiomicsFeaturesMaps.csv. The first-order Kurtosis feature value was incremented by 3 to be compliant with its Image Biomarker Standard Initiative (IBSI) definition^34^.

#### Step 5: Output format conversion

We utilized open source converters included in the *dcmqi* library^35^ to save segmentation results and radiomics features as DICOM Segmentation (SEG) and DICOM Structured Reports (SR) following template TID1500 “Image measurements’’, respectively. *TotalSegmentator* structure labels were mapped to the SNOMED-CT^36^ terms, per DICOM conventions. Conversion of the NIfTI to DICOM SEG was done using the *dcmqi itkimage2segimage* tool parameterized with the source DICOM CT series for the propagation of the composite context^37^ and mapping from the labels encountered in the NIfTI to SNOMED-CT terms. To enable conversion of the *pyradiomics* extracted features into DICOM SR, individual feature names were mapped to the IBSI terminology^34^, followed by the application of the dcmqi *tid1500writer* tool for conversion. Resulting files were compressed using *lz4* and transferred to a cloud bucket.

The workflow above was first implemented as a Jupyter notebook^38^ using Google Colab - a cloud-hosted Jupyter notebook service developed and hosted by Google - resulting in a fully functional prototype available in *CloudSegmentator* workflows/TotalSegmentator/Notebooks/endToEndTotalSegmentatorNotebook.ipynb. Next, we parameterized the resulting notebook using the open source Papermill^39^ tool. Papermill makes it possible to parameterize and execute python notebooks, thus allowing their use for batch analysis. In our case, the resulting notebook was parameterized with the list of the DICOM series to be processed.

### Deployment and optimization of the analysis workflow on CRDC Cloud Resources

Once the functional prototype of the workflow was finalized in the form of a Jupyter notebook, we proceeded with experimental deployment of the workflow in the Terra and SB-CGC environments. All of the components discussed in this section are available within the accompanying *CloudSegmentator* source code repository^31^ (https://github.com/ImagingDataCommons/CloudSegmentator).

First, we prepared Docker images containing the tools needed by the workflow. We defined several variants of the images containing different subsets of the pipeline steps. This was done to evaluate the impact of scheduling individual tasks of the workflow between GPU and CPU VM configurations on the overall cost. Only the task containing *TotalSegmentator* inference can benefit from the GPU. Scheduling individual tasks of the single workflow instance across different VMs will, however, result in additional data transfers and scheduling overheads. Most cloud providers offer *preemptible* or *spot* VMs, which are available at significant discounts as compared to the standard VMs, but can be stopped at any time by the hosting service based on the usage demand. Executing all of the tasks on a single preemptible VM will increase the overall uninterrupted time requirement, and will lead to increased possibility of interruption. We designed different configurations of the workflow to enable experimental evaluation of these options. For each of the workflow configurations below, we defined a Docker image (see *CloudSegmentator* workflows/TotalSegmentator/Dockerfiles) and a Python notebook (see *CloudSegmentator* workflows/TotalSegmentator/Notebooks):

1. **oneVM** (shown in Figure 1B): all steps of the workflow are executed as a single task, which is executed on a single VM as is usually done in a traditional pipeline.
2. **twoVM** (shown in Figure 1C): the workflow steps are split into two tasks: 1) downloading the DICOM images, converting those into NIfTI format, and performing TotalSegmentator inference (steps 1–3 of the pipeline), and 2) radiomics feature extraction and conversion of the inference results (steps 4–5). Tasks can be executed on two different VMs.
3. **threeVM**: the workflow is split into three tasks: 1) input download and format conversion, 2) inference, 3) radiomics feature extraction, and output format conversion. Each of the tasks can be executed on a separate VM.

Next, we prepared definitions of the analysis workflows using WDL and CWL to enable their execution on the Terra (see *CloudSegmentator* workflows/TotalSegmentator/Terra/splitWorkflow) and SB-CGC platforms (see *CloudSegmentator* workflows/TotalSegmentator/SevenBridges/splitWorkflow), respectively. Conceptually, both WDL and CWL workflow definitions are composed of one or more tasks. For workflows consisting of multiple tasks, Terra/SB-CGC can automatically route the outputs of an intermediate task as inputs for the subsequent task. The definition of each task includes the following components:

1. **runtime**: VM environment for executing the task, which includes CPU, GPU, RAM, memory, and storage requirements, and the Docker image that will be used to create the container to execute the task. Both SB-CGC and Terra make it possible to prescribe the use of preemptible VMs, which may be stopped (preempted) at any time. While Terra can restart a job with another preemptible VM up to a number defined by the configuration parameter, SB-CGC always restarts the preempted job with a regular non-preemptible VM.
2. **command**: instructions defining the processing steps that will be performed in a given task. In our case, each of the tasks is defined by a Python notebook and is executed and parameterized using Papermill.
3. **input** and **output**: variables parameterizing execution of a given task and capturing the results.

The choice between the CPU or GPU platform for executing the individual tasks can be controlled from the workflow definition, which makes it easy to evaluate trade-offs related to the use of GPU or preemptible VMs.

Once the components above are established, it is possible to use the workflows either via the notebooks or by deploying in the Terra or SB-CGC environments from the Dockstore^40^ interface (see https://dockstore.org/organizations/ImagingDataCommons/collections/CloudSegmentator). In both cases, the workflow is parameterized with the list of DICOM CT series selected from IDC and defined by their SeriesInstanceUID identifiers.

### Considerations for cloud-based workflow execution

Execution of a workflow in a cloud environment is highly configurable, contributing to both its flexibility and complexity. The ability to predict, bound and minimize the costs of workflow cloud-based execution is critical. In this section, we discuss some of the major configuration options that impact cloud costs. The following are based on our observations primarily from Google Cloud, although most of them may be generalizable to other cloud providers.

#### VM configuration

Cloud VMs can be configured with different classes of CPU families. Most of the AI models can benefit from GPU accelerators for inference. Cloud providers offer a variety of CPU and GPU architectures, but limited guidance on optimizing cost/performance trade-offs. Preemptible, or spot VMs are offered at a 50–90% (https://cloud.google.com/spot-vms) discount compared to their non-preemptible counterparts. Both Terra and SB-CGC support preemptible VMs and can automatically restart tasks that were preempted. Embarrassingly parallel computations, such as segmentation of the individual DICOM series, can be adapted to tolerate preemptions by optimizing the size of the batch and number of restart attempts.

#### Egress

While most cloud providers do not charge for *ingress* - the movement of data into their cloud environment - almost all charge for *egress* - the movement of the data out of the cloud. Furthermore, egress costs may be encountered even when the data moves between different zones or regions within the same cloud provider. As of this writing, depending on the selection of the source and destination regions while moving the data even across the resources of the same cloud provider, egress costs can vary between $0.01 and $0.23/GB^41^. Such charges may be unexpected to the users who are new to the cloud and may be significant. It is therefore very important to confirm the understanding of the egress costs associated with a given computational workflow and optimize the latter to reduce egress costs to the minimum possible with a given cloud provider.

#### Storage

Cloud-based solutions provide a multitude of data storage services that differ based on their performance and pricing characteristics, ranging from the VM-attached Solid State Drive (SDD) and Hard Disk Drive (HDD) storage to archival-grade storage buckets optimized for infrequent access. Again, tradeoffs can be difficult to navigate while configuring a computational workflow. The choices of a specific storage solution may have implications on the costs of other services (e.g., slower storage may lead to longer processing times, and increasing costs associated with VM tenancy). Among different classes of attached disk storage, traditional HDD is the cheapest. To optimize for the costs, and since the *TotalSegmentator* or radiomics feature extraction is not IO-intensive, we chose HDDs over SSDs.

#### Compute Regions

Users of the cloud need to be cognizant of the concept of *compute region*, which defines geographical location of the VMs. Pricing of cloud resources varies across cloud compute regions. Determining optimal selection of such resources is not straightforward, since GCP does not provide a comprehensive overview of pricing of the VMs or GPUs across regions, and such pricing is dynamic. To help with estimating pricing for a specific region, Google provides a Pricing Calculator tool (https://cloud.google.com/products/calculator) and an API (see https://cloudpricingcalculator.appspot.com/). We leveraged the API and utilized a notebook *CloudSegmentator* util/pricingOptimization/Top_20_cheapest_GPUs.ipynb), to survey pricing across regions and to guide compute region selection. It is important to note that the pricing API operates under the assumption that compute resources are available in every region. In practice, we found it necessary to manually confirm resource availability in each region via the GCP console.

#### Quotas

To reduce the possibility of extreme cost overruns, cloud providers utilize safeguards known as *quotas* - maximum allocation limits for the specific cloud resources, or the use of specific APIs. Users with appropriate permissions can either request modifications to the default quota values, or modify those directly (depending on the specific quota).

#### API usage costs

The use of API-based cloud-based services incurs usage costs. Any use of APIs at scale should be preceded by a careful evaluation to understand these costs. It is important to recognize that API costs are at times offset by the “free tier” allowance. Specifically, in the context of accessing data from IDC, one may utilize BigQuery for data selection directly from the processing workflow. As of writing, BigQuery queries are free as long as the amount of the processed data is within 1 TiB in a given month (see https://cloud.google.com/bigquery/pricing#free-tier). Our implementation utilized BigQuery prior to workflow execution to select DICOM series suitable for analysis, as shown in Figure 1. To enable lookup of the files corresponding to the DICOM series defined by SeriesInstanceUID we utilized the *idc-index*, which packages key DICOM metadata attributes and can be used as a cost-free alternative to BigQuery for basic search operations. Workflow performance optimization

To evaluate the performance of different workflow configurations, we captured or calculated the following utilization metrics and compared them between workflow execution on Terra and SB-CGC.

#### CPU, GPU, Disk

To assess the efficiency of resource allocation, we monitored the utilization of CPU, RAM, and GPU during the analysis. This information from initial observations was then used to assign the minimum necessary number of vCPUs, and size of RAM.

#### Egress

Our preparatory analysis showed that the cost of the NVIDIA T4 GPU located in the same region as the Terra workspace bucket (us-central1) was prohibitive (50% more than in other locations such as australia-southeast1, us-west4, asia-east1, asia-northeast1, and europe-west2). To explore the tradeoffs between GPU cost savings and egress charges transferring data across regions we performed experiments applying “twoVM” and “threeVM” configurations to small subsets of data. We calculated the egress charges using a combination of FISS API (https://github.com/broadinstitute/fiss) and processing billing logs stored in Google’s bigquery for Terra (https://support.terra.bio/hc/en-us/articles/360037862771-How-much-did-my-workflow-cost). On SB-CGC, we utilized the *sevenbridges-python* API (https://github.com/sbg/sevenbridges-python) to analyze costs separately in each of the compute, storage, and egress categories.

#### Preemption rate

To assess the frequency of VM preemptions, we calculated the preemption rate, which we defined as the total number of preemptions divided by the expected number of VMs required to perform a task in a batch. We also analyzed the distribution of these preemptions by task, along with the duration of allocated instances running uninterrupted before being preempted.

#### Wall Clock time

We defined wall clock time differently for the cohort and individual batches (see Table 1 for reference). *cohortWallClockTime* was defined as the time from the submission to the completion of analysis. On the other hand, *batchWallClockTime* was defined as the time from the time the workflow platform queuing the batch for processing to the completion of the processing (see Table 1). This distinction was necessary due to the peculiarities of the workload management systems of both Terra and SB-CGC, which limit the maximum number of workflows that can be executed at the same time.

#### Resource allocation/deallocation times

Although there is no official documentation on the workflow execution stages in Terra, we found 19 distinct values of status reported by the FISS API, while SB-CGC API lists only 4 stages. We normalized these timestamps as shown in Table 1. We defined *allocationAndSetupTime* as the time from job acceptance by the platform until the necessary input files for job execution are localized. The *coreAnalysisTime* was defined as the duration of executing the Colab notebook is executed by Papermill, and *deallocationTime* as the time from the conclusion of the core analysis until the platform marks the job as finished.

#### Processing workflow execution logs

To determine the optimal resource allocation strategy, we assessed and compared the costs associated with each configuration type (“oneVM”, “twoVM” and “threeVM”). We then separated the costs by task and investigated the contributions to the overall cost by the individual components (i.e., GPU, CPU, RAM, egress, etc.). Terra’s FISS API and SB-CGC *sevenbridges-python* API were used to retrieve workflow and task metadata for the workflows executed on the respective platforms. Both APIs report costs as well at the task level. Terra provides an option to access billing logs via BigQuery (https://support.terra.bio/hc/en-us/articles/360037862771-How-much-did-my-workflow-cost). Using SQL queries, billing data was used to retrieve costs associated with the individual workflows, tasks, and cloud components (CPU, GPU, RAM, etc). Plots were generated either using *matplotlib* and *seaborn* Python packages, and *Tableau*. For post-processing of the workflow metadata, we used a colab notebook (*CloudSegmentator* workflows/TotalSegmentator/Notebooks/PostProcessingTerra.ipynb) with Docker-based local run time (https://research.google.com/colaboratory/local-runtimes.html) on a JetStream2^42^ VM (available via ACCESS^43^ credits allocation) with 8 vCPUs 30 GB RAM.

#### Exploration of the analysis results

Once the computation was completed, we set up the environment to interactively explore and visualize the resulting dataset. We retrieved and decompressed artifacts generated by individual batches (DICOM SEG and SR objects containing analysis results) from the Terra workspace bucket, uploaded the resulting objects to a Google Cloud Storage bucket, and ingested them into a Google HealthCare API DICOM store. Metadata from all DICOM objects was extracted and exported to a BigQuery table using Google Healthcare API DICOM Store BigQuery export functionality. OHIF (Open Health Imaging Foundation) viewer^44^ (https://github.com/OHIF/Viewers) in combination with the Google Looker Studio dashboard were used to visualize and examine the generated results for each of the cohorts during the incremental development.

### Comparison between the cost of on-demand GCP VMs and on-premises analysis

In order to put the cloud-based performance in perspective, we estimated time and cost of performing the analysis of the final cohort using the computational resources available within our institution - Mass General Brigham (MGB) Enterprise Research InfraStructure (ERIS). Characteristics of these computational resources and pricing available for the members of the institution are publicly available at https://rc.partners.org/about/who-we-are-risc/enterprise-research-infrastructure-services. ERIS provides access to 5 GPU-equipped nodes, each with 8 NVIDIA V100 GPUs that are priced at $0.01/min/GPU (see https://rc.partners.org/kb/article/3650). In addition, CPU-only cluster contains 5 nodes with a total of 328 cores and 1.8 TB RAM. The CPU nodes are available free of charge (see https://rc.partners.org/kb/article/4036).

## Results

Our cloud-based analysis of the NLST cohort employed a staged approach, starting with a subset of 1037 series (the cohort established earlier by Krishnaswamy et al.^32^, further referred to as “1k cohort”), followed by 10,000 series (“10k cohort”) and ultimately to all eligible series in NLST (“126k cohort”). We scaled the analysis in stages so that we could identify and remediate deficiencies in our workflow before analyzing the entire cohort and experiment with the various configuration choices that were not trivial. Further, this staged approach allowed us to mitigate cost overruns and detect unexpected situations (e.g., workflows that are in a non-responsive state) while running the workflow on the cloud. While evaluating the various configuration options for executing the workflow and refining our approach leading to the final experiment, we utilized a combination of empirical choices for some of the parameters and conducted a more principled comparison for others. We selected this pragmatic strategy given the overwhelming number of options available to the user while performing large-scale computation on the cloud.

Our final implementation is available under a permissive license in this GitHub repository: https://github.com/ImagingDataCommons/CloudSegmentator. We used release v1.2.0 of the software^31^ for the final processing of the NLST collection. We made a collection of our CWL and WDL workflows available in Dockstore (https://dockstore.org/organizations/ImagingDataCommons/collections/CloudSegmentator), accompanied by the brief instructions for importing the workflows to a platform of the user’s choice in our GitHub repository.

### Evaluation of the input selection query

We conducted a systematic evaluation of the SQL query efficacy in filtering out the problematic image series by selecting cohorts of increasing sizes as follows. The SQL query underwent refinements based on insights gained from each experiment.

#### 1k cohort:

This cohort was selected using the baseline query introduced in a prior study by Krishnaswamy et al,^32^ which resulted in 1037 series selected (further referred to as “1k cohort”). 2 (0.19%) series bypassed the SQL query filters but failed the *dcm2niix* conversion step due to inconsistent slice intervals, prompting adjustment of the query. Conversion of certain JPEG-compressed series (TransferSyntaxUID of ‘1.2.840.10008.1.2.4.70’ and ‘1.2.840.10008.1.2.4.51’) resulted in failures. The query was then adjusted to exclude such problematic series by incorporating the TransferSyntaxUID DICOM attribute. Note that the original query by Krishnaswamy et al. included only cancer-positive patients from NLST. This restriction was removed in the revised query for building the subsequent cohorts.

#### 10k cohort:

This cohort of 10,000 CT series was formed by applying the revised query and including all 3,249 series which had more than 300 slices – to stress the GPUs and resource allocation. The remaining 6,751 series were selected randomly. All series were successfully processed without any failures at the NIfTI conversion step.

#### 126k cohort:

**Figure 2:**
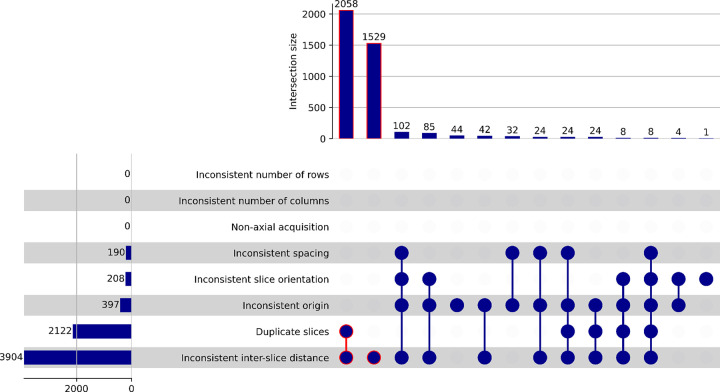
UpSet plot summarizing the combinations of the characteristics of the DICOM series from the NLST collection that were identified to have inconsistent geometry. Histogram on the top shows the distribution of the series that have the combinations of characteristics identified by the blue dots connected with the lines in the bottom right section of the plot. Histogram on the left summarizes the frequency of occurrence of the specfic geometric issues identified. The definition of the rules to identify these series was done by using an SQL statement (see CloudSegmentator workflows/TotalSegmentator/sqlQueries/nlstCohort.sql) against the DICOM metadata available in IDC. Items outlined in red correspond to the DICOM series groups constituting the largest portion of those that have inconsistent geometry.

**Figure 3:**
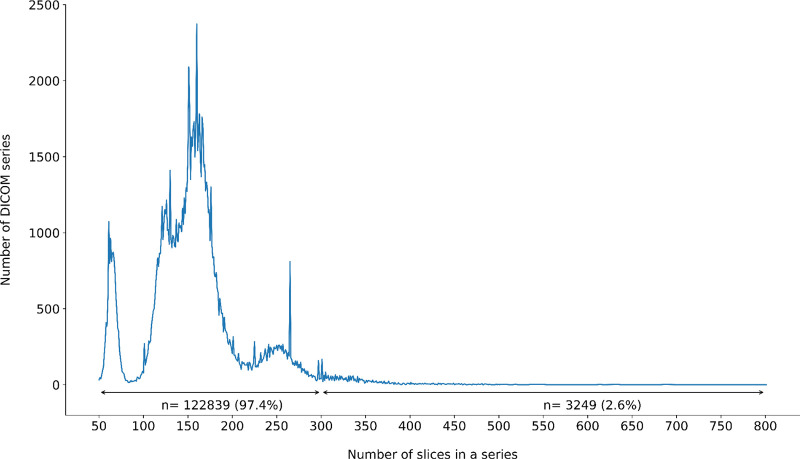
Distribution of the number of slices per DICOM series in the final 126k cohort used in the analysis. The tail of the distribution (n=3,249) corresponds to the DICOM series with the largest number of slices that were included in the 10k and 126k cohorts.

As of IDC release v17, NLST collection contains a total of 203,087 CT series from 73,113 studies corresponding to 26,254 patients^14^. We removed series identified as localizers (65,181/203,087, 32.1%), JPEG compressed series (161/203,087, 0.1%), series with missing ImagePositionPatient attribute (3/203,087, 0.002%) and series with less than 50 slices (7,669/203,087, 3.78%). Further, 1.96% of the series (3,985/203,087) were eliminated due to inconsistent geometry. The contributions of various problematic series are summarized in Figure 2. The final cohort included 126,088 series from 71,661 studies corresponding to 26,194 patients, with the majority of the series containing between 100 and 350 slices (122,839/126,088, 97.4%), see Figure 3. While processing this selection, 35 series failed the *dcm2niix* conversion. Four of those failed due to gantry tilt warnings, while the remaining 31 series exhibited inconsistencies in ImageType values within a series. One series encountered failure due to missing pixel data in two DICOM files.

### Overall optimization of the VM configuration and workflow execution

Before conducting any of the computational experiments we studied the available choices for CPU and GPU architectures and the selection of the compute regions to optimize the time/cost performance of the computation, as discussed below.

#### Virtual Machine configuration

Since the performance of a VM depends on the CPU architecture, we chose the most efficient VMs available on the platform. On Terra, we chose the N2D VM class (based on the AMD-Rome CPU architecture) for CPU-only tasks. The N2D VM class has a CoreMark (https://www.eembc.org/coremark/) score of 1.46 (1 vCPU), the highest among the CPU classes available on Terra (N1, N2, and N2D). On the other hand on SB-CGC we chose the c5 VM class – based on Intel Cascade Lake CPU architecture. N2D had the best price to performance among the options on Terra, and c5 was the most efficient among the AWS instance options on SB-CGC (c4, c5, m4, m5, r4, and r5). In addition, concurring with the suggestions available in the Terra documentation (https://terra.bio/speed-up-your-machine-learning-work-with-gpus), we found that the NVIDIA Tesla T4 GPU was the most cost-efficient among the GPUs available on Terra (K80, P4, P100, T4, and V100) and SB-CGC (T4 and V100). Initial observations showed that for series with greater than or equal to 300 slices, the amount of memory in the 4 vCPU16 GB RAM VM configuration was not sufficient for extracting radiomics features using multithreading. Whenever a batch contained a series that had 300 or more slices, an 8 vCPU and 32 GB RAM VM was assigned (see *CloudSegmentator* workflows/TotalSegmentator/Notebooks/Preprocessing.ipynb).

#### Configuration of resources for parallel workflow execution

Prior to each of the experiments we assessed the pricing of the resources needed for computation across the available cloud regions, and adjusted the selection based on the availability. Access to GPU resources within individual regions is controlled by a quota, which for the T4 GPUs had the default value set by Google Cloud at 16 for each of the regions. Once the desired compute regions were identified for a given experiment, we proceeded to request quota increases (between 500 and 1,024 from the default quota values) for those regions to allow for increased parallelism of the computation. While using Terra, this quota increase has to be requested through the GCP customer support interface and is subject to review by the GCP administrators (https://support.terra.bio/hc/en-us/articles/360029071251-How-to-troubleshoot-and-fix-stalled-workflows). SB-CGC users are required to submit such quota increases via the SB-CGC customer support and not directly to AWS.

It is important to note that the maximum possible parallelism of the workflow execution is also bounded by the limit of 3,000 workflows (in our implementation, each batch corresponds to a separate workflow) at a time on Terra (https://support.terra.bio/hc/en-us/articles/360055105051-Overview-How-the-workflow-system-works). Similarly, SB-CGC has a quota limit of 80 batches at a time (https://docs.cancergenomicscloud.org/docs/about-task-execution#queueing).

#### 1k cohort: Comparison of Terra and SB-CGC

To guide selection of the optimal platform and workflow configuration, we evaluated “oneVM”, “twoVM”, and “threeVM” on both Terra and SB-CGC, considering four key factors: cost, quotas, preemption rate, and analysis time.

The initial experiment of processing the 1k cohort was performed on June 8, 2023 (dates are important to allow for their consideration in the context of the pricing on the specific date).

**Figure 4:**
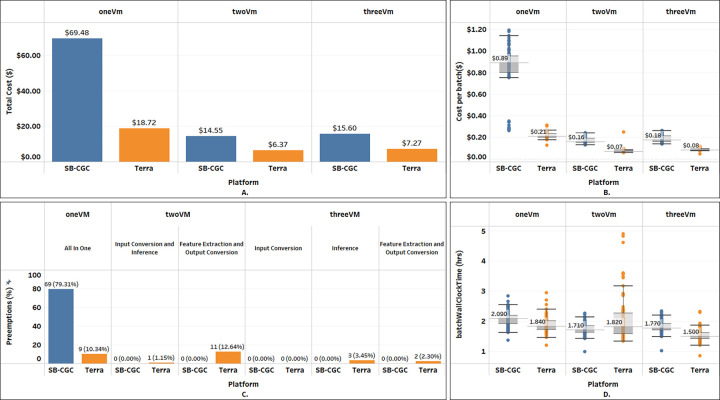
Summary of the key metrics analyzing the 1k cohort across Terra and SB-CGC platforms using the three VM configurations. **A**: total cost; **B**: cost per batch; **C**: VM preemption rate (%) for the individual configuration-specific processing steps; **D**: batch wall clock time. Boxplot shows median value, gray rectangle corresponds to the range of 25th to 75th percentile, whiskers are 1.5*IQR, and colored dots correspond to the data points and include minimum and maximum values.

**“twoVM”** emerged as the most cost-effective among all configurations on both Terra and SB-CGC, as summarized in Table 2 and Figure 4. Terra achieved lower overall costs at $6.37, with a 56.2% cost reduction in comparison to SB-CGC. The median cost per 12-series batch on SB-CGC was $0.16 - more than double that of Terra at $0.07. On Terra, the processing was interrupted by preemptions during Input Conversion and Inference once, and 11 times during the Feature Extraction and Output conversion (see Figure 4C). However, on SB-CGC no preemptions occurred during either of the steps. The median *batchWallClockTime* of 1.82 hrs on Terra was slightly higher than 1.71 hrs on SB-CGC (see Figure 4D).

**“threeVM”** ranked second, incurring costs only slightly higher than “twoVM”. Terra demonstrated an overall 53.4% cost reduction over SB-CGC (see Figure 4A) and a lower per-batch processing cost. Compared to “twoVM”, the “threeVM’’ configuration was 14.1% more expensive on Terra ($7.27 vs $6.37) and 16.6% more on SB-CGC ($15.60 vs $14.55). Similar to the “twoVM” experiment, we observed a small number of preemptions on Terra but not on SB-CGC. The median *batchWallClockTime* of 1.50 hrs on Terra was slightly lower than 1.77 hrs on SB-CGC.

**“oneVM”** was the least optimal in terms of costs and wall clock time to complete the analysis of the 1k cohort. Overall cost savings performing the analysis on Terra were even more prominent, with a 73.1% cost reduction compared to SB-CGC (see Figure 4A). The median cost per batch on SB-CGC of $0.89 was more than four times that of $0.22 on Terra (see Figure 4B). The preemption rate increased for both platforms, but this time was much higher for SB-CGC: over 79% vs 10.34% on Terra (see Figure 4C). The median *batchWallClockTime* of 1.84 hrs on Terra was slightly lower than 2.09 hrs on SB-CGC.

**Figure 5:**
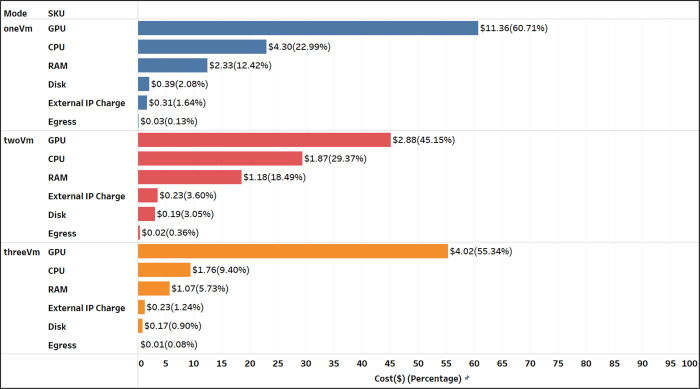
Breakdown of the overall costs by the individual Stock Keeping Units (SKU) items for the 1k cohort analysis experiment on Terra.

Following our analysis of the 1k cohort, we opted for the “twoVM” configuration and Terra platform for the subsequent experiments. This choice was largely influenced by the cost-efficiency, particularly that of GPUs, despite the higher preemption rate of the “twoVM’’ configuration (see Figure 5) and comparable processing time on Terra compared to SB-CGC. In addition, as mentioned previously, Terra’s 3000 batch limit quota is more suitable for scaling than the SB-CGC limit of 80.

#### 10k cohort: Refinement of the workflow

The 10k cohort analysis was performed on January 23, 2024. In preparation for the experiment, the regions for GPUs were updated to select the cheapest regions as of January 20, 2024, to account for the change in pricing by Google since the initial experiment. We utilized a heuristic that selected regions that were within 150% of the cost of the cheapest region, which resulted in the following regions: us-west4, us-east4, europe-west2, asia-northeast1, asia-southeast1, and europe-west4.

The 10k cohort was split across 834 batches for processing. Processing of 6 series in 6 batches failed due to insufficient robustness of the error handling during radiomics feature processing, leading to the aborted processing of the entire batch. As a result, a total of 72 series were not processed. Implementation was updated to improve robustness for the subsequent experiment.

**Figure 6.**
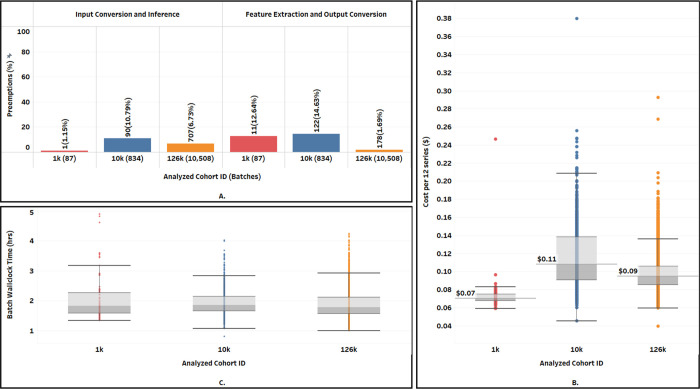
Comparison of the key performance metrics across the cohort sizes analyzed on Terra with the “twoVM” configuration. A: preemption rate (%); B: cost per batch (12 series); C: distribution of wall clock times (the time elapsed from the moment Terra/SB-CGC accepts the job until the job is finished).

The total cost of processing the 10k cohort was $97.23. This was 58.4% more than the projected cost from the 1037 cohort results: assuming linear scaling, the expected total costs would be $61.4, given the total costs of $6.37, or $0.00614/series, observed for the 1k cohort. The median cost per batch in the 10k cohort was $0.11 - higher than the median of $0.07 for the 1k cohort (see Figure 6). The preemption rate increased compared to the 1k cohort during Input Conversion and Inference (10.79% vs 1.15%) and similarly during Feature Extraction and Output conversion (14.63% vs 12.64%). Despite a higher preemption rate, analyzing the 10k series cohort finished in 4.02 hrs compared to 4.90 hrs for the 1k cohort due to increased parallelism. The total *coreAnalysisTime* for the 10k cohort was approximately 48 days while it was ~4 days for the 1k cohort (see Table 3). The median *batchWallClockTime* of 1.85 hrs for the 10k cohort was comparable to 1.82 hrs for the 1k cohort (see Figure 6).

Barring the aforementioned error handling issue there were no other major complications during scaling from 1k to 10k cohort. After adjusting the code, we launched the analysis for the entire cohort of 126,088 series.

### 126k cohort: Final analysis

Analysis of the final 126k cohort was performed on January 26, 2024. We split the cohort into 10,508 batches and allocated the compute resources the same way as we did for the 10k cohort. No batches failed completely but 35 series failed the *dcm2niix* conversion. There were no other processing errors.

The total cost for the 126k cohort was $1,011 which was 82.5% of the projected cost from the 10k cohort results (given the cost of $97.23 for 10,000, or $0.00097/series, the projection would be $1,226). The median cost per batch in the 126k cohort was $0.10 (see Figure 6). The preemption rate decreased as compared to what we observed in the 10k cohort both during Input conversion and inference (707/10,508 or 6.73% vs 10.79% for the 10k cohort), Feature Extraction and Output Conversion (178/10,508 or 1.69% vs 14.63% for the 10k cohort). Analyzing the entire 126k series cohort finished in 8.22 hrs with a total core computation time of ~522 days (see Table 3). The median batch wall clock time of 1.79 hrs for the 126k cohort was slightly lower compared to 1.85 hrs in the 10k cohort (see Figure 6).

**Figure 7:**
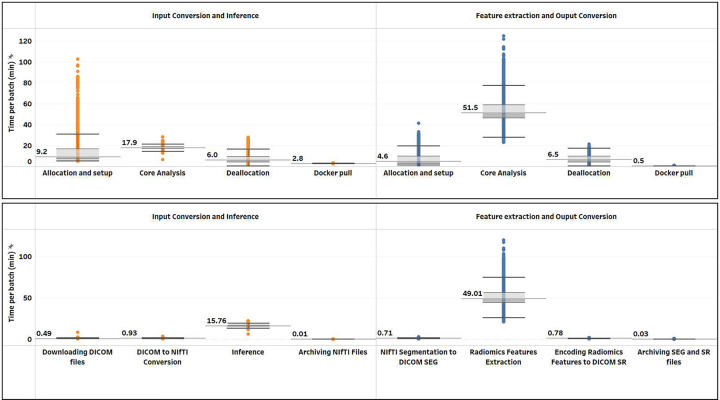
Distributions of the individual per-batch processing step times for the 126k cohort (10,508 batches) analysis on Terra using the “twoVM” configuration. See further details in Table 4 Top: allocationAndSetup, coreAnalysis, deallocation, and Docker pull (included in allocation time) times; Bottom: breakdown of the core analysis step into further substages within each task. One outlier batch was excluded for both plots. Deallocation time includes copying output files from the VM to the Terra workspace bucket, releasing the VM, and updating Cromwell’s job store.

An in-depth analysis of wall clock time components, as shown in Figure 7 and Table 4, revealed interesting patterns. On average, the time to allocate a GPU VM for the inference step (9.2 min) was higher than that of the CPU VM allocation in the subsequent step (4.6 min). *coreAnalysisTime* was the main contributor to the *batchWallClockTime*. Note that in the inference task allocation/deallocation time (~15 min median) was comparable with the core processing time (18 min median), which may justify further investigations for the selection of a more optimal batch size. The Radiomics feature extraction step appears to be the main contributor to the overall processing time (over 72% of the median cumulative core analysis time of ~68 min). Conversion of data to and from DICOM representation adds a negligible amount of time to the overall processing.

**Figure 8:**
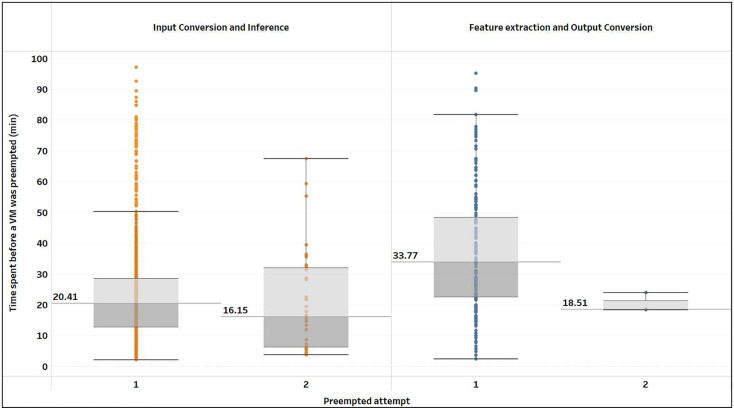
Summary of the computational time lost due to VM being preempted as a function of the processing stage and preemption event. Summary show corresponds to the processing of the 126k cohort using the “twoVM” configuration on Terra.

Preemptions of the VMs result in reduced efficiency in the use of computational resources since the analysis performed up to the preemption will be discarded. We investigated the frequency of preemptions and the time lost (see Figure 8 and Table 5). Overall, the total number of preemptions was <10% relative to the total number of batches. Out of those batches that were preempted, over 90% were preempted only once, with the median time to preemption (i.e., computation time lost) of 20.4 and 33.8 min for the inference and feature extraction stages, respectively.

**Figure 9:**
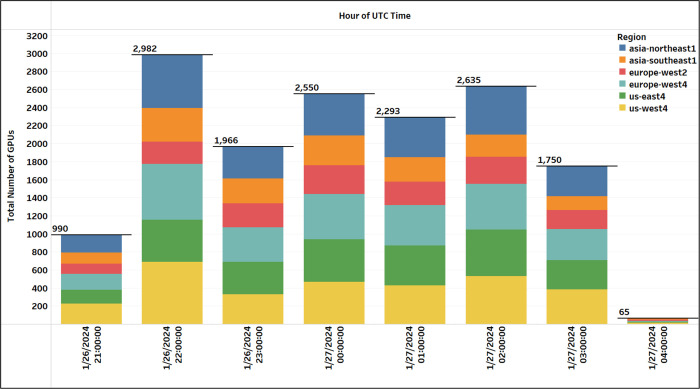
Summary of the cross-region GPU allocation for the final cohort analysis. Allocation is summarized at one-hour intervals since the launch of the 126k cohort (10,508 batches) processing using “twoVM” configuration on Terra.

Execution logs provide a wealth of information to investigate various aspects of the processing. Figure 9 provides a detailed depiction of GPU allocation (excluding the preempted attempts) by Terra over the 8.22 hours of processing the 126k cohort in 10,508 batches. The graph reveals that a minimum of 1,750 GPUs were consistently in use from the second hour through the seventh hour of the computation. The peak GPU usage was observed in the second hour at 2,982 GPUs. By the final hour, the GPU count had significantly reduced to 65, coinciding with the majority of batches transitioning into the Feature Extraction and Output Conversion task.

**Figure 10:**
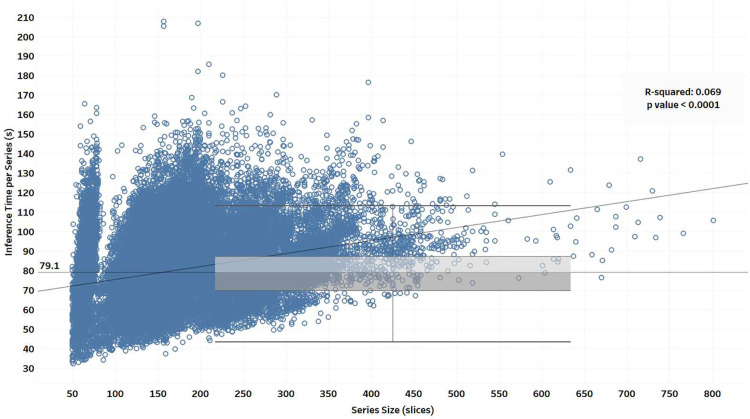
Distribution of DICOM series size and the inference times in the 126k cohort. Investigation of the factors that led to the observed multimodal distribution is of interest.

Figure 10 shows the relationship between the *TotalSegmentator* inference time and the number of slices in a given CT series. The inference time was under 2 minutes for most of the series, with a median of 79 seconds (IQR 69–87; range 32–207). There was a significant correlation (p<0.0001) between the number of slices and inference time.

**Figure 11:**
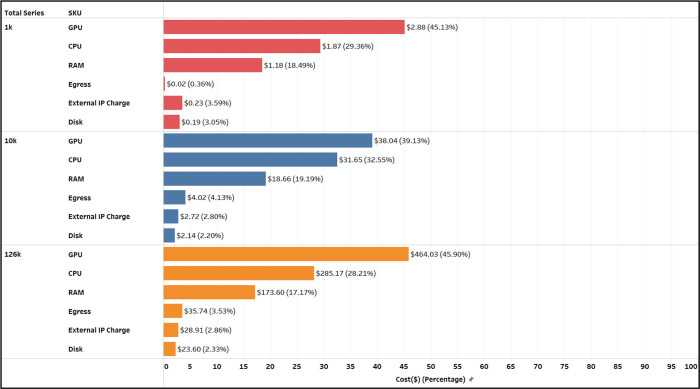
Breakdown of the major cost contributors for the performed analysis experiments. The summary corresponds to the major computational components across the cohort sizes analyzed using the “twoVM” configuration on Terra.

We examined the cost contributions of various compute components across the three cohorts (see Figure 11). Despite the increase in GCP prices between the 1k cohort and subsequent experiments, the cost contributions of the six components - GPU, CPU, RAM, egress, external IP charge, and disk - remained relatively consistent across all three experiments. As anticipated, the GPU was the largest contributor, accounting for 39–46% of the total costs. The CPU and RAM followed, contributing approximately 30% and 20% respectively.

### Review of the analysis results

Terra limits the kind of operations one can do outside of running workflows. We could not create DICOM stores in the GCP project created by Terra. Therefore, we decompressed the DICOM SEG and DICOM SR objects generated from Terra into Google Cloud Storage buckets as outlined in the methods section. This step took approximately 4 days for the 126k cohort. We could have parallelized and sped up this operation by developing another workflow on Terra but it was not part of this experiment. Next, we used Google Healthcare API to import the data from the buckets into a DICOM store. The DICOM metadata was then exported into a BigQuery table for further exploration. It is noteworthy that the process of importing data into the DICOM store from Storage buckets and exporting DICOM metadata for the final cohort took less than an hour. We deployed an instance of the OHIF Viewer^44^ to connect to the resulting DICOM store to facilitate a review of the segmentation results. We also prepared a Google Looker Studio dashboard, which exposed a small subset of DICOM metadata available in the aforementioned BigQuery table to enable examination of the segmentation results.

The setup discussed above was used to examine analysis results produced during different stages of development.

### Comparison between the cost of on-demand GCP VMs and on-premises analysis

Since the T4 GPU architecture we used in the cloud-based analysis is not offered by ERIS, we made an assumption that V100 GPU is twice as efficient as T4, the cost of performing the computation on ERIS would be comparable to that on Terra. On Terra, it took a total of ~3,150 hours of *coreAnalysis* time for Input Conversion and Inference. At 200% efficiency on V100, and assuming the entire cluster is reserved, it would have taken ~1,575 hrs or *cohortWallClockTime*of 1 day 15 hrs among the 40 GPUs. At $0.01/min/GPU this would cost ~$945. CPU-only nodes at ERIS are available as shared resources, and can be used free of charge. Feature Extraction and Output Conversion step’s *coreAnalysisTime* on Terra took 8,271 hrs for 4 vCPU 16 GB RAM instances and 1,118 hrs for 8 vCPU 32 GB RAM instances. Based on this, we project that ERIS CPU-only cluster with 5 nodes and a total of 328 cores would have taken 5 days and 8 hrs ((8,271*4+1,118*8)/328). Note that these estimates do not account for any possible delays due to scheduling or waiting for the availability of these shared resources.

## Data availability

**Figure 12:**
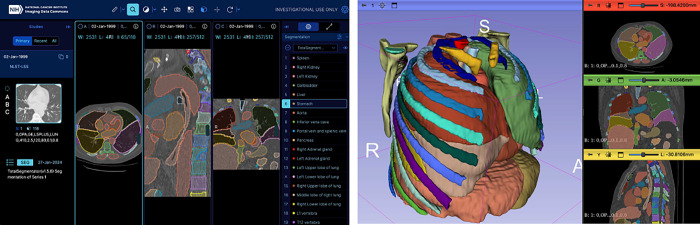
Visualization of a sample CT series and the corresponding segmentation. The case shown is for NLST PatientID 100004, time point 0. Left: Visualization using OHIF v3 viewer integrated within IDC Portal, as can be accessed at https://viewer.imaging.datacommons.cancer.gov/v3/viewer/?StudyInstanceUIDs=1.2.840.113654.2.55.174144834924218414213677353968537663991&SeriesInstanceUIDs=1.2.840.113654.2.55.195946682403058845904768502826466194287,1.2.276.0.7230010.3.1.3.313263360.11967.1706318547.125050. Right: Visualization of the same CT and SEG series using 3D Slicer 5.6.2 (SlicerIDCBrowser extension was used to download data from IDC, QuantitativeReporting extension was installed to support loading of DICOM SEG into the Slicer scene).

The final set of the analysis results was deposited into IDC as a new *TotalSegmentator-CT-Segmentations* analysis results collection^45^ and is available for interactive exploration at https://portal.imaging.datacommons.cancer.gov/explore/filters/?analysis_results_id=TotalSegmentator-CT-Segmentations. Manifests corresponding to the files containing the results along with the instructions for download are available as part of the data descriptor accompanying this analysis results collection^45^. Figure 12 shows visualization of a sample result.

## Discussion

In this study, we demonstrated the potential of Terra and SB-CGC platforms, and cloud computing in general, for automating AI-based curation applied to large datasets, on the example of the TotalSegmentator segmentation model applied to the NLST collection. While AI is not a substitute for human expertise in all annotation tasks, at least some applications, such as imaging-based population studies, image region-based feature extraction for cross-omics analyses, or content-based retrieval, can benefit from the automatic volumetric annotations. To our knowledge, there is no practical alternative to obtain annotations of the anatomy generated in this study utilizing human experts at this scale.

Our comparison of the costs and time of performing the presented analysis using on premises resources demonstrates that while costs may be comparable with the cloud, the scale of resources available, at least at our institution, is not. It is important to note that our estimates are arguably biased towards the on premises option by making somewhat unrealistic assumptions, such as complete access to the entire cluster for 1.5 days. It might be of interest to conduct experimental evaluation of executing the developed workflow using on premises resources. The workflows developed - in principle - should be interoperable with the cluster batch scheduling systems.

The choice between on premises and cloud-based analysis is complex. This study aims to demonstrate the capabilities of cloud analysis for a specific use case. Considerations for other use cases will vary from ours. At the same time, cloud-based analysis may be the only option for the researchers who do not have the luxury of access to on premises clusters, which require significant effort to maintain and upgrade. Cloud-provisioned computational resources can help improve equity for researchers and institutions that cannot afford their own local computational facilities. On the other hand they also provide great flexibility for researchers who have the financial resources to take advantage of cloud computing features. In addition, institutions that currently invest heavily in on premises computing may be able to offer more cost-effective and adaptable computing services via specialized cloud providers in comparison to traditional methods, although there are many considerations beyond the price/performance computing issues explored in this study.

While the process of developing the workflow may appear daunting from the lengthy description provided in this preprint, the initial implementation was completed by an engineer (VKT) who did not have prior experience with either medical imaging, Google Cloud Platform, Terra or DICOM within just a few months (under the guidance and with the support of the more experienced members of the team). Further, with the source code of *CloudSegmentator* accompanying this report, we attempt to simplify the process of utilizing CRDC CRs for alternative segmentation tasks. We plan to introduce additional segmentation tools into the repository, and improve usage instructions and examples.

To date, Terra and SB-CGC platforms have primarily been used for genomics applications, with no publicly available examples of utilizing them for cancer imaging research. Both platforms operate as computational sandboxes fostering team collaboration while providing granular control over permissions. By making workflow methods publicly accessible either through publishing on Dockstore or enabling access on their respective platform app stores it becomes possible to not only simplify access to the complex analysis tools, but also promote transparency and reproducibility, and encourage further innovation in the field by allowing others to replicate and improve upon these methods.

In comparing Terra and SB-CGC platforms – within the scope of the present study – both are capable of running end-to-end workflows and allocating compute resources to individual tasks with varying requirements. However, Terra emerged as the more cost-efficient and flexible option in our initial 1k cohort experiment across all three configurations of the workflow execution that we examined. Terra offers a wider range of VM options, allows retrying a preempted task with another preemptible VM, and can allocate compute resources from any GCP region. It also provides access to detailed billing reports allowing us to gain insights about spending and optimize costs, allows modifications of the quota limits without interacting with the technical support. In addition, it has a superior data model that conveniently organizes output file locations for downstream processing. In contrast, SB-CGC’s abstraction limits the flexibility, restricts users to a single compute region, restricts access to billing and quotas, and requires the use of the ‘sevenbridges’ API for data organization. We note that those users who have a technical background will likely appreciate Terra’s granularity, while those with limited technical experience might prefer SB-CGC’s ease of development aided by the GUI and automatically written CWL code. Overall, Terra’s advantages make it our clear choice for large-scale analysis.

In our comprehensive assessment of the considered workflow configurations, the “twoVM” approach on Terra stood out as a reliable and cost-effective solution, something that was particularly noticeable during the incremental scaling of the processed cohort size. Our experiments underscored Terra’s scalability and cost efficiency. It is perhaps not surprising that the “oneVM” is the least cost-effective configuration since GPU use contributes significantly to the overall costs while being only utilized during the Inference step. While *TotalSegmentator* can run on a CPU, our initial results indicated that it is several times slower, and therefore it is more expensive in total than using a VM with a GPU. We did not run the full cohort to explore the cost/performance tradeoffs for various GPU types, but based on initial experiments we chose the NVIDIA T4 as a reasonable option for the full cohort experiment. Using preemptible VMs significantly reduces the overall costs for both CPU and GPU instances. The results from the “threeVM’’ configuration experiments, however, illustrate an interesting aspect of the price/performance behavior of this workflow. It might appear that delegating the downloading of DICOM files and conversion to a non-GPU VM would be more cost-effective. However, our experiments demonstrated that the downloading and conversion of the images is so rapid that delegating this task to a CPU-only VM does not lead to cost savings.

Our selected toolset significantly enhanced efficiency in maintaining the code and optimizing costs. By using DICOM metadata interrogated via SQL queries we prevented wasteful attempts to process ~38% of the NLST image series that were unsuitable for the *TotalSegmentator* model. We achieved substantial cost savings by using cheaper preemptible VMs, dynamically allocating resources based on task and batch requirements, and selecting the most inexpensive GPUs globally. Our use of Colab notebooks and GitHub for version control streamlined codebase management. In combination with Papermill, this allowed us to keep WDL/CWL files lean, decouple notebook development, and generate output notebooks that included logs for troubleshooting. We also found DockerHub to be the most reliable, fast, and efficient option for hosting Docker images compared to other repositories (comparison against other container registries was not shown). *lz4*, a fast compression tool helped mitigate egress costs, compressing DICOM SEG files by over 90%. Linking GitHub to Dockstore helped maintain versioning and facilitated seamless integration of the CWL and WDL files. Finally, converting the artifacts generated by TotalSegmentator and the radiomics features into standardized DICOM format enabled interoperability with Google Healthcare API, which in turn simplified quality checks of the produced analysis results.

One may wonder about how generally applicable are the approaches and tools utilized in this study. Our workflows are currently suited for processing publicly available de-identified image data from IDC, with no restrictions on locations where data can be processed. While the infrastructure is perfectly suitable for processing non-public data, additional considerations must be taken into account. The data may need to be de-identified before the analysis on the Terra and Google Cloud resources. It is the responsibility of the user to ensure compliance with all the rules and regulations governing the data being analyzed. GPUs available outside the US may be priced more competitively, however, it may not always be possible to use regions outside of the US due to the governance restrictions applied to specific datasets. While our analysis utilized public GCP cloud resources, in principle, one could apply the developed workflows using private cloud or on premises compute resources.

Our workflows relied heavily on the availability of various managed services such as DockerHub for hosting Docker images and GitHub for the management of config files and jupyter notebooks. If there is a requirement to keep Docker images and Jupyter notebooks private, or hosted on cloud storage, costs may increase rapidly as cloud providers charge both for storage and egress when Docker images are downloaded. Our implementation is currently relying on Google services. To a degree, we mitigate the perceived risk of cloud vendor lock-in by utilizing standard interfaces for communicating with the cloud services. Data retrieval is done over S3 API for downloading files, and over DICOMweb while accessing DICOM data from the Google Healthcare API by the image viewers. BigQuery search operations are done using SQL.

Beginner users of Terra or SB-CGC aiming to develop new workflows will benefit from at least introductory knowledge of computing components, coding, Docker, and cloud. Jupyter notebooks are becoming ubiquitous in research. The transition from the notebook to Terra/SB-CGC will require some understanding of Docker technology. Containerizing the code and its dependencies may be challenging, particularly when access to the attached GPU is necessary. On the other hand, it should be rather easy to adjust workflows developed by others (including those that we shared), where containerization has already been completed. Once a workflow is refined, it will enable scalable analysis while keeping time and costs low by allocating computing resources as needed.

In the future, we plan to apply workflows like this to more of the images available in IDC and share the results publicly. Depending on the feedback from the community, we will consider extracting other types of radiomics features - beyond first order and shape - for the segmented structures. Optimization of radiomics feature extraction might be of interest given it is the main contributor to the overall processing time and cost. In the present study we did not investigate optimization of the batch size, which might further improve utilization of the resources. A more rigorous comparison of the cloud and on premises analysis and comparison of different cloud backends (AWS and Azure) is warranted.

## Figures and Tables

**Table 1:** Mapping between the terms used in Terra and SB-CGC and the nomenclature adopted in our study. List of timestamped events reported by Terra and SB-CGC during the lifetime of a task execution, and the mapping to the corresponding time intervals adopted in our study. Gray zones are the times that SB-CGC does not separate with the same precision as Terra does.

Terra task execution status	Equivalent SB-CGC task execution status			Nomenclature adopted in our study	
Pending	Duration	Queued duration		allocationAndSetupTime	batchWallClockTime
RequestingExecutionToken					
PreparingJob					
WaitingForValueStore					
RunningJob					
waiting for quota					
Worker “google-pipelines-worker-xxxxxx” assigned in {region} on a “custom-2-13312” machine		Running duration			
Pulling “gcr.io/google.com/cloudsdktool/cloud-sdk:354.0.0-alpine”					
Pulling “imagingdatacommons/{docker image tag}@sha256:xxxx”					
ContainerSetup					
Background					
Localization					
UserAction			Execution duration	coreAnalysisTime	
CheckingForMemoryRetry				deallocationTime	
Delocalization					
Worker released					
Complete in GCE / Cromwell Poll Interval					
UpdatingCallCache					
UpdatingJobStore					

**Table 2:** Summary of the processing times and costs for the evaluated workflow configurations across the Terra and SB-CGC platforms. Processing steps correspond to those in Figure 1: Step 1: Data retrieval, Step 2: Input conversion, Step 3: Inference, Step 4: Feature extraction, Step 5: Output conversion. Highlighted in green are the rows corresponding to the configuration/platform that demonstrated optimal performance in analyzing 1k cohort, and the final cohort processing.

Workflow conf.	Platform	Processing steps	Total cost, USD	Cohort	Batches	Cost per batch, USD median (IQR) [range]	batchWall ClockTime, hours median (IQR) [range]	cohortW allClockTime	Preemptions, %
oneVM	Terra	1–5	$18.72	1k	87	$0.22 (0.20–0.23; 0.13–0.31)	1.84 hours (1.71–2.00) [1.20–2.94]	2 hrs 57 min	9
**twoVM**		1–3	**$6.37**			**0.07 (0.07–0.08) [0.06–0.25]**	**1.82 hours (1.57–2.25; 1.33–4.89)**	**4 hrs 54 min**	**1**
		4–5							**11**
threeVM		1–2	$7.27			$0.08 (0.08–0.09; 0.05–0.12)	1.50 hours (1.41–1.61; 0.85–2.31)	2 hrs 19 min	0
		3							3
		4–5							2
oneVM	SB-CGC	1–5	$69.48			$0.89 (0.80–0.95; 0.26–1.19)	2.09 hours (1.91–2.19; 1.36–2.82)	3 hrs 48 min	69
twoVM		1–3	$14.55			$0.16 (0.15–0.19; 0.13–0.24)	1.71 hours (1.63–1.84; 0.99–2.25)	3 hrs 44 min	0
		4–5							0
threeVM		1–2	$15.60			$0.18 (0.16–0.21; 0.14–0.26)	1.77 hours (1.70–1.91; 1.02–2.33)	3 hrs 22 min	0
		3							0
		4–5							0
twoVM	Terra	1–3	$97.23	10k	834	$0.11 (0.09–0.14; 0.05–0.38)	1.85 hours (1.66–2.14; 0.81–4.02)	4 hrs 1 min	90
		4–5							122
**twoVM**	**Terra**	1–3	**$1,011.05**	**126k**	**10508**	**$0.10 (0.09–0.11; 0.04–0.29)**	**1.79 hours (1.56–2.11; 0.99–4.25)**	**8 hrs 13 min**	**707**
		4–5							**178**

**Table 3:** Summary of costs, cohort size, wall clock, and total compute times while scaling from 1k to 10k and ultimately to 126k series cohorts using twoVM Configuration on Terra. Note that total core analysis time excludes the time for allocation, setup, and deallocation of a VM, as summarized in Table 1.

Cohort size, DICOM series	Slices per series, median (inter-quartile range) [min-max]	Total size of the files processed	Virtual Machines used*	Cohort Wall Clock Time	Cumulative batch Wall clock time	Cumulative core analysis time**	Total Cost	Average Cost per series
1k	157 (39) [101–342]	89 GB	87 × 2	4 hrs 54 min	7 days 12 hr 43 min	4 days 1 hr 45 min	$6.37	$0.00614
10k	171 (107) [50–801]	1.23 TB	834 × 2	4 hrs 1 min	67 days 5 hrs 2 min	48 days 9 hrs 28 min	$97.23	$0.00972
126k	155 (47) [50–801]	10.61 TB	10,508 × 2	8 hrs 13 min	823 days 15 hrs 46 min	522 days 10 hrs 26 min	$1,011.05	$0.00802

* Each batch analyzed in the twoVM configuration required the use of a GPU equipped VM for the inference step and a CPU VM for the radiomics feature extraction step, thus the total number of the VMs used is twice the number of batches for each cohort, excluding preempted VMs.

** The results for 10k and 126k cohorts listed in Figure 10 presented in Fedorov et al.^7^ differ from the values in this table as workflow definitions and GCP pricing evolved since the earlier publication. The total compute time was incorrectly reported in that figure for the 126k cohort. While the cumulative coreAnalysis time was reported for 1k and 10k cohorts, for the 126k cohort cumulative batchAnalysis time was reported. The total compute time for the 126k cohort should have been reported as 460 days 6 hrs 19 min instead of 785 days 13 hrs 1 min.

**Table 4:** Contribution of the various components to the overall processing time. Highlighted rows correspond to the most time-consuming steps.

Workflow component (“twoVM” configuration)	Breakdown of *batchWallClockTime*		Breakdown of *coreAnalysisTime*	
	Stage	Time, minutes median (IQR) [range]	Step	Time, minutes median (IQR) [range]
Input Conversion and Inference	Allocation and setup	9.2 (7.0–16.5) [5.09–102]	N/A	
	Core Analysis	18 (17.0–18.8) [6.7–28.1]	Downloading DICOM files	0.49 min (0.36–0.72 [0.14–8.0]
			DICOM to NIfTI Conversion	0.93 min (0.77–1.12) [0.44–2.97]
			**Inference**	**15.76 min (0.77–1.12) [15.0–16.6]**
			*lz4* compression of NIfTI files	0.01 min (0.012–0.015) [0.005–0.03]
	Deallocation	6 (3.9–9.0) [0.5–27.8]	N/A	
Feature Extraction and Output Conversion	Allocation and setup	4.6 (2.8–9.5) [1.4–41.6]	N/A	
	Core Analysis	52 (46.3–58.8) [22.3–125]	Conversion of NIfTI segmentations into DICOM SEG	0.71 min (0.64–0.86) [0.29–2.47]
			**Radiomics Features Extraction**	**49 min (43.8–56.1) [20.7–120]**
			Conversion of radiomics features JSON into DICOM SR	0.77 min (0.72–0.87) [0.31–1.60]
			*lz4* compression of DICOM SEG and SR files	0.032 min (0.029–0.036) [0.013–0.147]
	Deallocation	6.5 (4.0–9.5) [0.5–21.5]	N/A	

**Table 5:** Summary of the preemptions observed during the 126k cohort analysis using the “twoVM” workflow configuration.

Task	Preempted Attempt	Preemptions	Time to preemption, min median (IQR) [range]
Steps 1–3 (Data retrieval, Input Conversion and Inference)	1	693	20.4 min (12.6–28.3) [2.1–97]
	2	39	16.2 min (6.2–32) [3.7–67)
	3	2	8.3 min [6.1–10.5]
Steps 4–5 (Feature Extraction and Output Conversion)	1	175	33.8 min (22.5–48.3) [2.4–95]
	2	3	18.5 min [18.3–24]
